# Analysis of l-glutamic acid fermentation by using a dynamic metabolic simulation model of *Escherichia coli*

**DOI:** 10.1186/1752-0509-7-92

**Published:** 2013-09-22

**Authors:** Yousuke Nishio, Soichi Ogishima, Masao Ichikawa, Yohei Yamada, Yoshihiro Usuda, Tadashi Masuda, Hiroshi Tanaka

**Affiliations:** 1 Institute for Innovation, Ajinomoto Co. Inc., Suzuki-cho 1-1, Kawasaki-ku, Kawasaki City, Kanagawa 210-8681, Japan; 2 Department of Bioinformatics, Tokyo Medical and Dental University, Yushima 1-5-45, Bunkyo-ku, Tokyo 113-8510, Japan; 3 Pharmaceutical Custom Manufacturing Department, Ajinomoto Co. Inc., Kyobashi 1-chome 15-1, Chuo-ku, Tokyo 104-8315, Japan; 4 Research Institute for Bioscience Products & Fine Chemicals, Ajinomoto Co. Inc., Suzuki-cho 1-1, Kawasaki-ku, Kawasaki City, Kanagawa 210-8681, Japan; 5 Faculty of Symbiotic Systems Science, Fukushima University, Kanayagawa 1, Fukushima City, Fukushima 960-1296, Japan; 6 Current address: Department of Health Record Informatics, Tohoku Medical Megabank Organization, Tohoku University, Seiryo-cho 4-1, Aoba-ku, Sendai-City, Miyagi 980-8575, Japan

**Keywords:** l-glutamic acid fermentation, Dynamic metabolic simulation, Sensitivity analysis, Phosphoglycerate kinase, *Escherichia coli*

## Abstract

**Background:**

Understanding the process of amino acid fermentation as a comprehensive system is a challenging task. Previously, we developed a literature-based dynamic simulation model, which included transcriptional regulation, transcription, translation, and enzymatic reactions related to glycolysis, the pentose phosphate pathway, the tricarboxylic acid (TCA) cycle, and the anaplerotic pathway of *Escherichia coli*. During simulation, cell growth was defined such as to reproduce the experimental cell growth profile of fed-batch cultivation in jar fermenters. However, to confirm the biological appropriateness of our model, sensitivity analysis and experimental validation were required.

**Results:**

We constructed an l-glutamic acid fermentation simulation model by removing *sucAB,* a gene encoding α-ketoglutarate dehydrogenase. We then performed systematic sensitivity analysis for l-glutamic acid production; the results of this process corresponded with previous experimental data regarding l-glutamic acid fermentation. Furthermore, it allowed us to predicted the possibility that accumulation of 3-phosphoglycerate in the cell would regulate the carbon flux into the TCA cycle and lead to an increase in the yield of l-glutamic acid via fermentation. We validated this hypothesis through a fermentation experiment involving a model l-glutamic acid-production strain, *E. coli* MG1655 Δ*sucA* in which the phosphoglycerate kinase gene had been amplified to cause accumulation of 3-phosphoglycerate. The observed increase in l-glutamic acid production verified the biologically meaningful predictive power of our dynamic metabolic simulation model.

**Conclusions:**

In this study, dynamic simulation using a literature-based model was shown to be useful for elucidating the precise mechanisms involved in fermentation processes inside the cell. Further exhaustive sensitivity analysis will facilitate identification of novel factors involved in the metabolic regulation of amino acid fermentation.

## Background

Understanding metabolic behavior in terms of a system is important for the design and fabrication of useful substances by biotechnological approaches [1]. Due to the progress in -omics studies and bioinformatics, including computer simulation, we can now integrate knowledge from different levels such as that pertaining to gene expression, protein expression, and metabolite concentrations. This information is useful for designing a strain for producing a particular substance [2]. Dynamic metabolic modeling is a useful approach for studying the regulation of metabolism by integration of biological knowledge from different biochemical systems. For example, mathematical modeling of the tryptophan operon in *Escherichia coli* clearly showed that TrpR and TrpL were responsible for high and low intracellular tryptophan concentrations, respectively [3]. This knowledge formed the theoretical basis for engineering a tryptophan-production strain [4].

l-glutamic acid, a flavor enhancer that is produced worldwide in quantities of over 2 million metric tons per year, is a very important fermentation product and is typically produced by fermentation using *Corynebacterium glutamicum*[5,6]. Studies on the mechanism underlying l-glutamic acid overproduction in *C. glutamicum* are in progress and have shown the importance of α-ketoglutarate dehydrogenase activity [7]. Additionally, *E. coli* has been used as a model microorganism for research and also as an industrial producer of useful substances, including amino acids and organic acids [8]. Specifically, the *E. coli* MG1655 Δ*sucA* strain, which lacks α-ketoglutarate dehydrogenase activity, has been used as a model l-glutamic acid-production strain [9,10].

To optimize carbon flux through gene deletion or gene amplification is one of the key technologies for production of substances based on fermentation. Several algorithms and methodologies have been proposed for the identification of a target for molecular breeding in metabolic engineering. Flux balance analysis is commonly used to obtain the theoretical maximum yield and the optimal biosynthesis pathway [11,12]. Additionally, OptKnock is used as a strategy for knocking out genes or pathways in order to find optimal biosynthesis pathways [13]. Elementary mode analysis is also used to analyze metabolic network analysis to obtain all possible combinations of reaction networks [14,15]. These approaches are based on a stoichiometric matrix of chemical reactions and static analyses. In contrast, dynamic simulation of cell metabolism is expected to become a useful method for analyzing and elucidating not only a metabolic state but also all transient in vivo cellular bioprocesses; such dynamic modeling requires knowledge of kinetic parameters.

There are mainly 2 approaches to obtaining kinetic parameters. One is parameter estimation, used for determining the coefficients in power-law equations utilizing the S-system [16]. The other involves obtaining the Michaelis constants and catalytic constants from biochemical experiments, in which Michaelis-Menten-type equations are used for modeling [17,18]. In a previous study, we constructed our own model for the glucose phosphotransferase system (PTS) in *E. coli*. It included the transcription of genes and their regulatory mechanisms, protein translation, and Michaelis-Menten-based approximations of enzymatic reactions, using parameters adopted from scientific literature. In a simulation study, this model indicated that amplification of *ptsI* would increase the specific glucose consumption rate; we subsequently validated this prediction experimentally [19]. We expanded this model and constructed a large-scale metabolic and regulatory model of *E. coli* central metabolism, which included the metabolic enzymes related to PTS, glycolysis, the pentose-phosphate pathway, the tricarboxylic acid (TCA) cycle, anaplerotic enzymes, and the glyoxylate shunt, as well as transcriptional regulation by the cyclic AMP receptor protein (CRP), making large colonies protein (Mlc), catabolite repressor/activator (Cra), pyruvate dehydrogenase complex repressor (PdhR), and acetate operon repressor (IclR) [10]. We also proposed a modeling approach to describe the successive transient phases in batch or fed-batch cultivation and successfully simulated the l-glutamic acid fermentation process using *E. coli* MG1655 Δ*sucA* as a model strain [10]. In this study, we improved our previous model, performed sensitivity analyses and simulation, and validated our model mathematically. This model led us to discover a new factor influencing l-glutamate production, which we then verified experimentally.

## Results

### Sensitivity analysis for l-glutamic acid production

Using a dynamic simulation model, we calculated the difference in l-glutamic acid production yields in response to variation in gene copy number. We defined the ratio of this difference to the variation in copy number as sensitivity. Because the sensitivity value changes depending on copy number variation, we obtained a maximum sensitivity value by changing the copy number variation from 0.001 to 1000. Moreover, we defined a scaling factor in Table 1 as the copy number that provides the maximum sensitivity value. The maximum sensitivity of l-glutamic acid production to the increase or decrease in copy number of 44 individual gene species is shown in Table 1; in this table, the top 15 genes (or operons) are arranged in descending order of maximum sensitivities. Based on this result, we classified the 15 factors that affected l-glutamic acid production in our model into 3 groups. The first group consisted of factors for which the enhancements directly involved the biosynthetic pathway of l-glutamic acid, including the sugar uptake system. The genes *icdA*, *gdhA, gltA*, *fba*, *tpiA*, *gapA*, *pgk*, and *pykF*, corresponding to the 1^st^, 4^th^, 5^th^, 6^th^, 8^th^, 11^th^, 12^th^, and 14^th^ items, respectively, in Table 1, encode the biosynthetic enzymes of l-glutamic acid. The genes *ptsG* and *crr*, corresponding to the 9^th^ and 13^th^ items in Table 1, respectively, encode components of the sugar-uptake system (PtsG and IIA^Glc^ of PTS). Enhancement of these factors is estimated to result in positive changes in l-glutamic acid production. In previous studies, an increase in citrate synthetase or glutamate dehydrogenase activity has been shown to be a factor leading to an increase in l-glutamic acid production in *C. glutamicum*[20-22]. Through sensitivity analysis, we confirmed a similar effect in *E. coli.* The maximum sensitivities for an increase in the copy number of *gltA*, which encodes citrate synthetase, and of *gdhA*, which encodes glutamate dehydrogenase, are 0.17565 and 0.21119, respectively (Table 1). Furthermore, in *E. coli*, we observed a 4.0 g/L increase of l-glutamic acid production in the *gltA* amplification strain and a similar 1.6 g/L increase in the *icdA* amplification strain in the fermentation experiment after 48 h cultivation (Table 2). These results suggested that there are similar metabolic principles for l-glutamic acid production in *E. coli* and in *C. glutamicum*.

**Table 1:** **Maximum sensitivity for l-glutamic acid production**

**No.**	**Gene or operon**	**Gene product**	**Maximum sensitivity**	**Scale factor**
1	*icdA*	isocitrate dehydrogenase	0.33615	1000
2	*aceBAK*	malate synthase, isocitrate lyase, isocitrate dehydrogenase kinase/phosphatase	0.27963	0.001
3	*iclR*	isocitrate lyase regulator	0.27693	1000
4	*gdhA*	glutamate dehydrogenase	0.21119	1000
5	*gltA*	citrate synthase	0.17565	1000
6	*fba*	fructose bisphosphate aldolase	0.09989	400
7	*pdhR*	pyruvate dehydrogenase complex regulator	0.09614	3
8	*tpiA*	triose phosphate isomerase	0.09399	4
9	*ptsG*	glucose-specific PTS permease, IICB domain	0.07520	500
10	*gpmA*	phosphoglycerate mutase	0.06702	0.001
11	*gapA*	glyceraldehyde-3-phosphate dehydrogenase	0.06605	600
12	*epd-pgk*	glyceraldehyde-3-phosphate dehydrogenase, phosphoglycerate kinase	0.06446	900
13	*crr*	glucose-specific PTS permease, IIA domain	0.05030	1000
14	*pykF*	pyruvate kinase	0.03785	5
15	*eno*	enolase	0.03391	0.001

**Table 2:** **L-Glutamic acid fermentation results of *gltA *and *icdA *gene amplification strain in *E. coli***

**Strain**	**OD**_**600**_	**l-glutamic acid**	**Residual**
		**Accumulation**	**Glucose**
		**(g/l)**	**(g/l)**
*E. coli* MG1655 Δ*sucA*/pTWV228-P_*tac*_-T_*trp*_	18.5 ± 0.2	16.6 ± 0.1	0.0 ± 0.0
*E. coli* MG1655 Δ*sucA*/pTWV228-P_*tac*_-*gltA*-T_*trp*_	16.2 ± 0.9	20.6 ± 0.8	0.0 ± 0.0
*E. coli* MG1655 Δ*sucA*/ pTWV228-P_*tac*_-*icdA*-T_*trp*_	16.1 ± 0.2	18.2 ± 0.2	0.0 ± 0.0

The second group of factors comprised those that decreased the carbon flux in the glyoxylate shunt, that is, *aceBAK* and *iclR*, which corresponded to the 2^nd^ and 3^rd^ items, respectively, in Table 1. The increase in *iclR* expression levels and the decrease in the *aceBAK* operon expression level led to a decrease in the carbon flux of the glyoxylate shunt. Furthermore, *icdA*, encoding isocitrate dehydrogenase, which caused maximum sensitivity, was also related to carbon flux distribution at the branch point of the TCA cycle and the glyoxylate shunt, indicating that the balance of the metabolic flux between the TCA cycle and the glyoxylate shunt is of critical importance for l-glutamic acid production.

For the third group of factors, including the *pdhR* (7^th^), *gpmA* (10^th^), and *eno* (15^th^) genes, it was difficult to determine the reason why decreased expression would lead to an increase in l-glutamic acid production. The reason why a 3-fold increase in the expression of *pdhR* resulted in maximum sensitivity for l-glutamic acid production is discussed later in the subsection “Perturbation analysis by *pykF* amplification.” Attenuation of the activity of phosphoglyceride mutase (GpmA) and enolase (Eno) led to a decrease in l-glutamic acid production, although both enzymes catalyzed reactions in the l-glutamate biosynthetic pathway. We assumed that a decrease in the expression of *gpmA* may lead to accumulation of 3-phosphoglycerate. A decrease in the expression of *eno* would also result in accumulation of 3-phosphoglycerate, since reactions catalyzed by GpmA and Eno are reversible. The detailed mechanism of the effect of 3-phosphoglycerate accumulation on glutamate production has been described later.

### Perturbation analysis by *pgk* amplification

**Figure 1:**
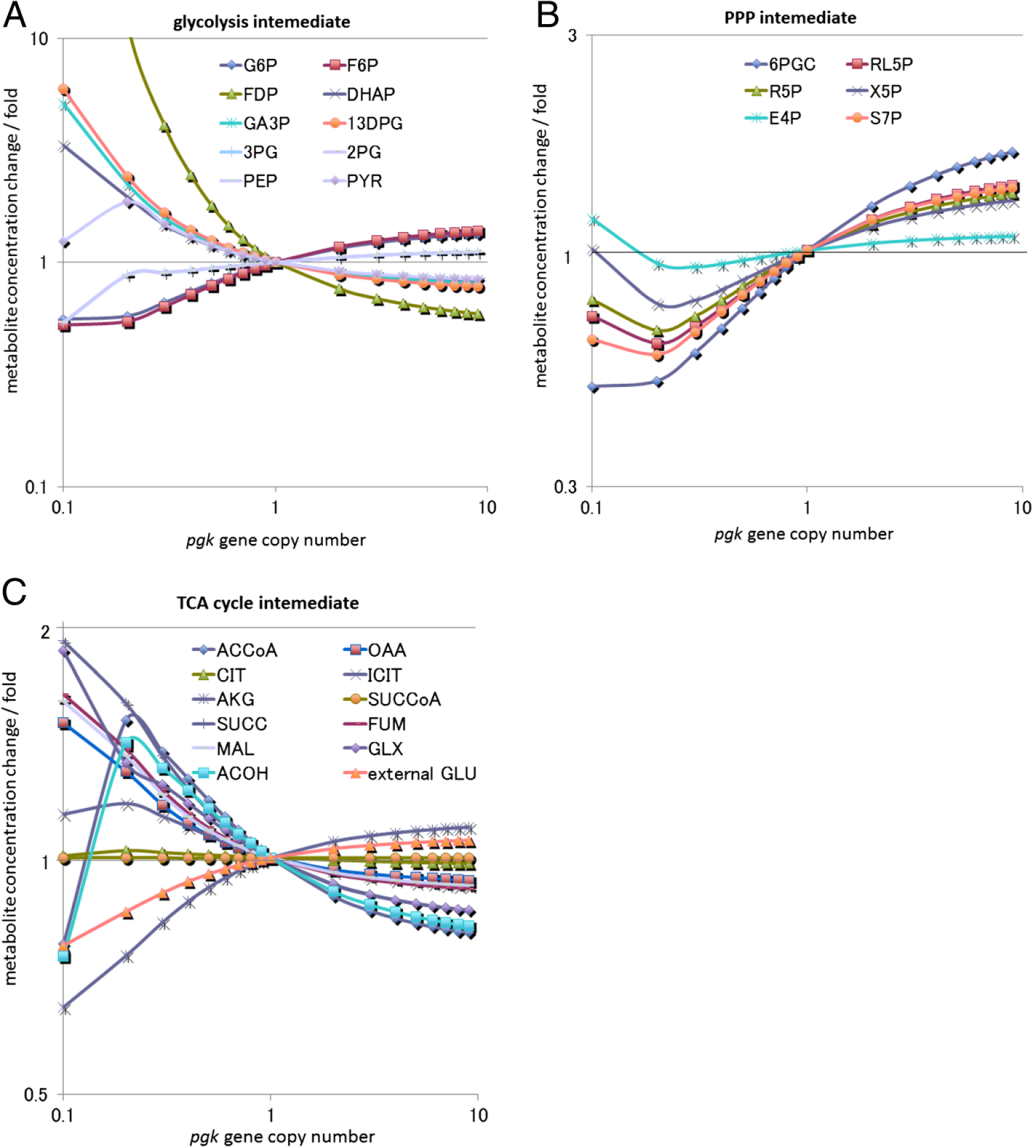
**Simulated results of perturbation of the *epd–pgk *operon.** The vertical axis in each graph shows the ratio of the concentration obtained for metabolites to the initial concentration of these metabolites; the horizontal axis shows the magnitude of perturbation. **(A)** Intermediates of the glycolysis pathway. **(B)** Intermediates of the pentose phosphate pathway. **(C)** Intermediates of the TCA cycle.

The top 5 entries in Table 1 are factors known to influence l-glutamic acid production. To identify novel factors using our simulation model, we analyzed the effects of enzyme concentration and gene copy number on l-glutamic acid production. In this analysis, we perturbed gene copy number by multiplying the copy number with factors from 0.001 to 1000 in a logarithmic manner. The results were plotted on graphs in which the horizontal axis corresponded to gene copy number and the vertical axis corresponded to l-glutamic acid and other metabolite concentrations. Through this analysis, we found that an increase in *pgk* copy number correlates with an increase in l-glutamic acid production (Figure 1).

**Figure 2:**
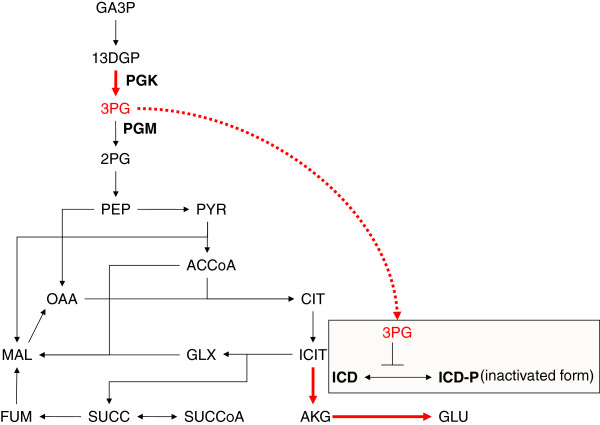
**Predicted mechanism underlying changes in l-glutamic acid production by *pgk *gene amplification.** A schematic representation of the simplified metabolic pathway from GA3P to l-glutamic acid. The effect of increased 3-phosphoglycerate concentration on phosphorylation of isocitrate dehydrogenase is indicated.

**Figure 3:**
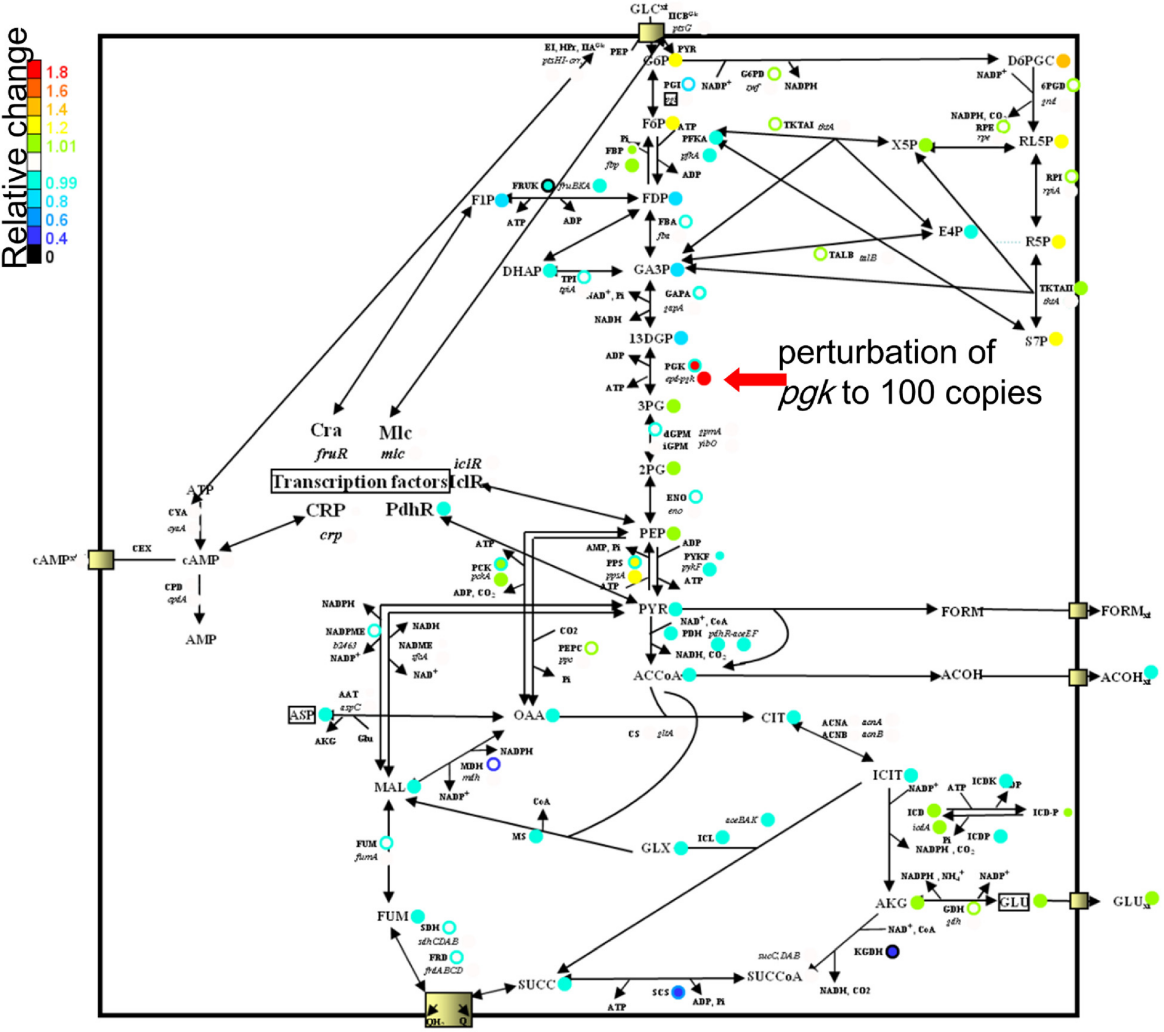
**Simulation of 100-fold amplification of the *pgk *gene.** Each color shows the relative change in concentration of each molecule. The circles next to the compound names indicate the relative change in concentration of the compound. The circles next to gene product names show the relative change in the concentration of the enzyme and mRNA encoded by the corresponding genes. The relative changes in mRNA and enzyme levels are presented in inner circles and outer circumferences, respectively.

Amplification of *pgk* was a novel important factor for l-glutamic acid production, and we attempted to interpret the mechanism underlying this observation. In *E. coli, pgk* encodes phosphoglycerate kinase. Phosphoglycerate kinase is known to catalyze the reaction converting 1,3-bisphosphoglycerate and ADP to 3-phosphoglycerate and ATP [23]. An increase in the *pgk* copy number would cause accumulation of 3-phosphoglycerate, which seemed to be closely correlated with the relationship between the decreased expression of *gpmA* and *eno* and accumulation of 3-phosphoglycerate. It is known that 3-phosphoglycerate acts as an inhibitor of isocitrate dehydrogenase kinase/phosphatase, which is encoded by *aceK* in *E. coli*[24,25]. Since unphosphorylated isocitrate dehydrogenase retains its activity, an increase in unphosphorylated isocitrate dehydrogenase is associated with an increase in l-glutamic acid production (Figure 2). In Figure 3, we have shown the accumulation of 3-phosphoglycerate and the consequential increase in l-glutamic acid production observed when simulating a100-fold amplification of *pgk*. If our model, simulation, and interpretation are appropriate, then the data indicate that more than 100 copies of *pgk* would increase l-glutamic acid production experimentally.

### l-glutamic acid production in a strain with amplification of *pgk*

To confirm the effect of *pgk* gene amplification in l-glutamic acid fermentation in *E. coli*, we constructed a *pgk*-expression plasmid by using the high copy-number plasmid pUC118. The gene *pgk* was cloned into this vector with its native regulatory sequence, promoter, and Shine-Dalgarno sequence [26]. The *pgk* expression vector was introduced into a model l-glutamate-producing strain, *E. coli* MG1655 Δ*sucA.* The results for l-glutamic acid fermentation are shown in Table 3. After 72 h of cultivation, no residual glucose was observed in all the strains. A 3.2 g/L increase in l-glutamic acid accumulation and a decrease in optical density at 600 nm by 2.0 were observed for cultures in which pUC118-*pgk* had been introduced, in comparison with those into which pUC118 had been introduced as a control. This result suggested that our hypothesis regarding the increase in l-glutamic acid production by *pgk* gene amplification was validated qualitatively.

**Table 3:** **l-Glutamic acid fermentation results**

**Strain**	**OD**_**600**_	**l-glutamic acid**	**Residual**
		**Accumulation**	**Glucose**
		**(g/l)**	**(g/l)**
*E. coli* MG1655 Δ*sucA*/pUC118	7.0 ± 0.4	16.2 ± 0.7	0.0 ± 0.0
*E. coli* MG1655 Δ*sucA*/pUC118-*pgk*	5.0 ± 0.5	19.4 ± 1.0	0.1 ± 0.3
*E. coli* MG1655 Δ*sucA*/RSF-PPG /pUC118	10.2 ± 0.6	20.8 ± 2.8	0.0 ± 0.0
*E. coli* MG1655 Δ*sucA*/RSF-PPG/pUC118-*pgk*	9.2 ± 0.9	22.1 ± 2.8	0.2 ± 0.4

During sensitivity analysis, an increase in *gltA* and *gdhA* expression levels was predicted to lead to an increase in l-glutamic acid production (Table 1). In a previous study, the RSF-PPG plasmid, containing the genes *ppc*, *prpC,* and *gdhA* was used in l-glutamic acid fermentation by *Pantoea ananatis,* a member of the *Enterobacteriaceae* family [27-29]. The genes *ppc* and *gdhA* encode PEP carboxylase and glutamate dehydrogenase, respectively, and *prpC* encodes citrate synthase activity in addition to methylcitrate synthase activity [30]. By introducing the RSF-PPG plasmid into *E. coli* MG1655 Δ*sucA*/pUC118, a 4.6 g/L increase in l-glutamic acid accumulation was observed, in comparison with a strain into which pUC118 had been introduced as a control (Table 3). This result qualitatively supported our sensitivity analysis results. After72-h cultivation, there was almost no residual glucose in the medium. By introducing the pUC118-*pgk* plasmid into *E. coli* MG1655 Δ*sucA*/RSF-PPG, a 1.3 g/L increase in l-glutamic acid accumulation and a decrease of 1.0 in the optical density at 600 nm were observed, when compared with *E. coli* MG1655 Δ*sucA*/RSF-PPG (Table 3). This experimental result suggested that our hypothesis regarding *pgk* amplification was also valid in a higher l-glutamic acid-accumulating strain and that the increase in l-glutamic acid production was at least partially due to a decrease in biomass production.

### Perturbation analysis by *pykF* amplification

**Figure 4:**
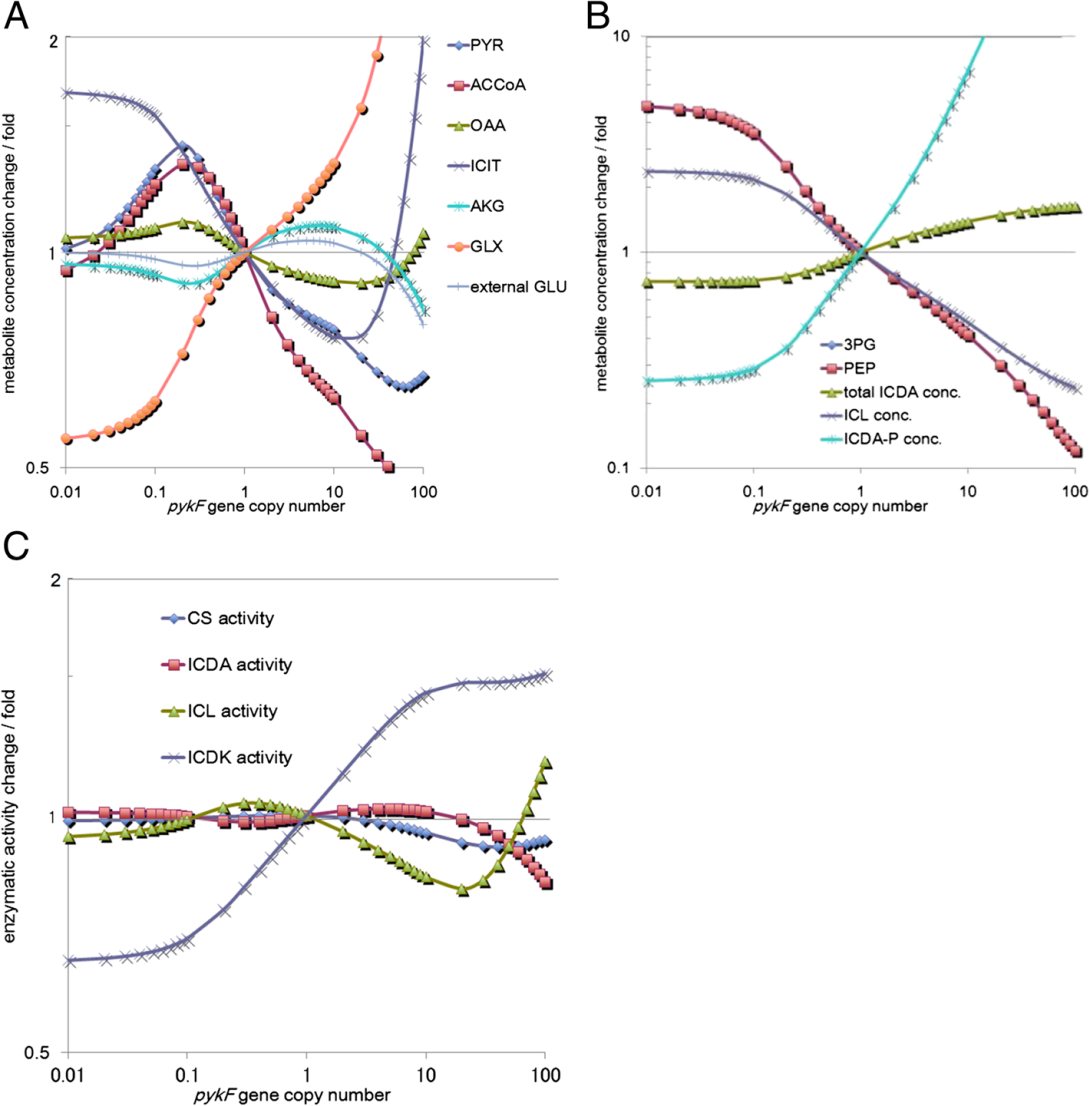
**Simulation of *pykF *perturbation.** The vertical axis in each graph shows the ratio of the concentration obtained for metabolites to the initial concentration of these metabolites or the ratio of enzymatic activity obtained to the initial enzymatic activity, while the horizontal axis shows the magnitude of perturbation. **(A)** Metabolites with a change rate between 0.5 and 2 fold. **(B)** Metabolites with a change rate between 0.1 and 10 fold; data for 3PG and PEP overlap. **(C)** Enzymatic activity change.

An interesting phenomenon was observed when we perturbed the copy number of *pykF*, which encodes pyruvate kinase. A maximum increase in l-glutamic acid production was observed at 5-fold amplification of the *pykF* copy number (Table 1). Further enhancement of the *pykF* copy number led to a decrease in l-glutamic acid production. We propose that the mechanism underlying these findings involves the following. On increase in the *pykF* copy number, the conversion of pyruvate to PEP increases, resulting in a decrease in the acetyl-CoA supply from pyruvate and an increase in glucose incorporation via glucose PTS (Figure 4A). The decrease in acetyl-CoA supply was closely related to the decrease in isocitrate supply (Figure 4A). As a result of *pykF* amplification, which raised PEP consumption, the concentration of 3-phosphoglycerate would be decreased, which would lead to increased inactivation of isocitrate dehydrogenase by the activation of isocitrate dehydrogenase kinase (Figure 4B, C). Our simulation study suggested that, even though the amount of the activated form of isocitrate dehydrogenase was reduced, the carbon flux toward α-ketoglutarate could be slightly enhanced by reduction of the carbon flux into the glyoxylate shunt when the isocitrate concentration is low. As a result, the l-glutamic acid production would increase. When the copy number of *pykF* is amplified more than 5 times, it leads to a decrease in 3-phosphoglycerate concentration, while most of isocitrate dehydrogenase would be inactivated (Figure 4B). As a result, l-glutamic acid production would be decreased. We suggest that this is why moderate enhancement of *pdhR* expression could have the same effect on pyruvate concentration as *pykF* amplification.

## Discussion

In our previous study, we constructed a dynamic simulation model of *E. coli* based on biological knowledge and reproduced the experimental cultivation results by parameter fitting [10]. In this study, we attempted to elucidate novel factors that affect l-glutamic acid fermentation by using dynamic simulation based on a computer-aided rational design of biochemical networks. First, we refined the model with respect to biomass production through model validation. Then, a precise sensitivity analysis was performed and revealed many factors that would be important for l-glutamic acid fermentation. For example, an increase in the expression of *gltA*, which encodes citrate synthase, *icdA*, which encodes isocitrate dehydrogenase, and a combined increase in the expression of both these genes were predicted to have a high impact on l-glutamic acid production. In fact, an increase in the expression of *gltA* or *icdA* enhanced l-glutamic acid production in *E. coli* (Table 2). These genes have already been utilized to optimize l-glutamic acid fermentation in an industrial strain, *C. glutamicum*[20,21], thus supporting the accuracy of our dynamic simulation model for understanding *E. coli* metabolism. In a previous study, we proposed that the putative transcriptional regulator YdcI controls carbon flux into the TCA cycle in *E. coli*[31]. We observed that an increase of citrate synthase activity by deletion of *ydcI* led to an increase in l-glutamic acid production, and a decrease of citrate synthase activity due to *ydcI* amplification led to a decrease in l-glutamic acid production. Our sensitivity analysis results support a clear relationship between YdcI and l-glutamic acid production, because both sensitivity analysis and experimental results clearly showed that a change in citrate synthase expression levels exert a significant effect on l-glutamic acid production in *E. coli*. Integration of the transcriptional regulator YdcI into our dynamic simulation model is a subject for future studies.

In theoretical flux analysis, the metabolic flux distribution, which facilitates the maximal theoretical yield of l-glutamic acid in *E. coli*, indicated that flux through α-ketoglutarate dehydrogenase (encoded by *sucAB*) and the glyoxylate shunt pathway (encoded by *aceBA*) should be 0. Thus, to achieve maximal l-glutamic acid production from the MG1655 Δ*sucA* strain, deletion of the *aceBAK* operon, which encodes enzymes in the glyoxylate shunt pathway and isocitrate dehydrogenase kinase/phosphatase, or amplification of *iclR*, which encodes a negative regulator of the glyoxylate shunt, would be a plausible approach. In contrast, using the dynamic simulation model in addition to sensitivity analysis, we identified unexpected factors. This approach indicated that amplification of *pgk* and attenuation of *gpmA* and/or *eno* would induce accumulation of 3-phosphoglycerate, which inhibits the phosphorylation of isocitrate dehydrogenase, and consequently results in increased glutamate production. Furthermore, the effects of amplification of *pykF* or *pdhR* on l-glutamate production could be explained according to a different mechanism. This type of working hypothesis cannot be generated using conventional theoretical flux analysis because it requires that the modification systems that control enzyme activity be taken into account. Thus, a critical advantage in using the dynamic simulation model is to be able to take various modes of regulation into consideration simultaneously and comprehensively.

In biotechnology, both production yield and productivity are important. In general, it is not simple to maintain productivity while improving production strains because the achievable yield and productivity can vary, depending on the strains and production conditions. From an engineering point of view, improving yield or productivity from a current production strain is a practical issue. One of the key features that affect productivity is biomass formation. In this study, we improved our simulation model to describe biomass production as precisely as possible; however, it was not sufficiently accurate to predict productivity. In *E. coli*, the carbon flux through the TCA cycle is known to affect biomass production directly [32]. Our dynamic simulation predicted the changes in concentrations of each molecule with response to gene amplification or deletion, and based on simulation results, we could speculate whether these perturbations would affect cell growth through carbon flux into the TCA cycle. In this study, we predicted that the concentration of 3-phosphoglycerate could play an important role in controlling the carbon flux through the TCA cycle; we subsequently validated this hypothesis experimentally. We also estimated that metabolic regulation through 3-phosphoglycerate would contribute to the changes in biomass production and fermentation productivity. In future, we plan to describe these changes more precisely in our model.

Our model requires further refinement. When we perturbed the copy number of *ppc*, which encodes PEP carboxylase, the sensitivity was 0.02138 and the ranking of this gene was 17^th^. In a previous study on *C. glutamicum,* it had been experimentally shown that an increase in PEP carboxylase activity led to an increase in l-glutamic acid yield, with a reduction in organic acid byproducts [33]. We speculated that one of the reasons for the low sensitivity of *ppc* was related to the process of parameter tuning because the catalytic constant of PEP carboxylase was modified to 100 times greater than the reported value. Thus, an increase in PEP carboxylase was not sensitively related to l-glutamate concentration in our model [10]. However, there is a difference in anaplerotic pathway enzymes and their regulation in *E. coli* and *C. glutamicum*[34]. Together with these facts, we speculate that unknown mechanisms, related to PEP carboxylase or an anaplerotic pathway in *E. coli*, could exist and result in inadequate modeling. The citrate synthase reaction requires both oxaloacetate and acetyl-CoA as substrates. An increase in PEP carboxylase expression will intensify the carbon flux toward oxaloacetate. However, to enhance citrate synthase activity, acetyl-CoA would be required. In our simulation model, the current acetate production model may be too simplistic to describe the dynamic changes in acetyl-CoA concentration inside the cell because the regulation of acetate metabolism, including formation, excretion, uptake, and utilization of this substance, is quite complex in reality [10]. In the future, improvement in simulating the metabolism of acetate, including its excretion, is a priority for refining our model.

We recognize that our model is currently limited in terms of quantitative predictive power. According to our sensitivity analysis, amplification of *pgk* would increase glutamate production yield by 106%, whereas, experimentally, we found a 120% increase. One of the factors that affect the prediction seems to be the difference between the experimental conditions considered for simulation and those used for validation. In the simulation model, data for the parameters were obtained using jar fermenters; however, experimental validation involved the use of shake flasks [10]. We assume that the most important issue related to the predictive power is the modeling of cell growth. In l-glutamic acid production using *E. coli* MG1655 Δ*sucA*, the succinyl-CoA used for biomass formation should be supplied through the glyoxylate shunt. Amplification of *pgk* in *E. coli* MG1655 Δ*sucA* decreases the carbon flux toward the glyoxylate shunt pathway. Consequently, we observed 2 phenomena, viz., increased l-glutamic acid production and decreased biomass production. At present, this trade-off has not been accommodated in our simulation because the cell growth profile is fixed in accordance with experimental results obtained using jar fermenters [10]. To further refine our model, the cell growth profile should be allowed to be variable, based on the concentration of biomass precursor molecules. Experimentally verified biological evidence will continue to be appropriately incorporated into our model during further refinements.

## Conclusions

In this study, we evaluated a literature-based dynamic metabolic pathway model of *E. coli* by computational analysis and verified it experimentally. Our kinetic metabolism model was particularly useful for analysis of feedback regulation systems, and it was stable and robust against perturbation. In future, we would need to improve cell growth modelling to improve the flexibility and quantitative prediction capability of the current model. This resource will contribute to metabolic engineering that predicts the key factors for substance production.

## Methods

### Model and modification of biomass production

Model construction is described in Usuda et al. [10]. The enzymatic reactions and transport processes were modelled based on Michaelis–Menten-type velocity equations. Gene expression was described by transcription and translation rate equations. The systems parameters and metabolite concentrations used as constants in the simulation were obtained largely from the literature. The initial values for metabolites, messenger RNA (mRNA), and proteins were set as described [10]. The parameters used for the enzymatic reactions, gene expression, and proteins levels were basically adopted from the literature. Promoter concentrations, rate constants of transcription, and mRNA degradation rates were estimated. The most important feature was that the RNA polymerase (RNAP) and ribosome concentrations were expressed as a function of the specific growth rate, μ, which varied during batch cultivation and was calculated from experiments.

We prepared a summation model for biomass formation, which comprised the consumption and production steps of key precursor substances required for biomass production. The key precursor substances and their quantitative composition reflected the actual biomass composition, and the biomass per gram was expressed as a summation of key precursor substances with stoichiometry coefficients [35]. This approach is well established in theoretical flux analysis [36], and we applied this methodology in our dynamic simulation model. Biomass formation was taken into account, based on the cell growth and the stoichiometric matrix, by subtracting the required amount of biomass formed from the precursor metabolite. In other words, if the stoichiometric coefficient of a key precursor of biomass composition is a positive value, the key precursor is used for biomass production, whereas if the stoichiometric coefficient of a key precursor is a negative value, the production of the key precursor is associated with biomass production. However, the choice of key precursor substances in this case would not be the same as in theoretical flux analysis [10]. In our previous model, the amounts of OAA and FUM molecules required for producing 1 g of biomass achieved negative values. This indicated that cell growth causes intracellular accumulation of OAA and FUM, which would not be in accordance with the metabolic process that we expect in *E. coli* and may have resulted in deviations from the experimental observations. Therefore, we revised the model so that the coefficients of OAA and FUM for producing biomass attained positive values.

We observed that lipid composition influenced the coefficients of OAA in our model. Odd-numbered fatty acids, as a part of biomass production, were set to 0 because, typically, fatty acids are synthesized as multiples of 2, and production of odd-numbered fatty acids is negligibly low. Saturated and unsaturated fatty acids with carbon numbers of 13, 15, and 17 were subjects for modification of our model. In a previous model [10], the FUM required for biomass production was itself produced not only from the TCA cycle, but also through the arginine biosynthesis pathway and the nucleotide biosynthesis pathway, as follows:

eq.A)ARGSUC‒>ARG+FUM

eq.B)SAICAR‒>AICAR+FUM

Because energy supply through the TCA cycle is primarily important for biomass formation and the stoichiometry coefficient of FUM in the previous model had a negative value, we assumed that the FUM produced via the steps shown in eq. A and eq. B was immediately and completely oxidized through the TCA cycle. We therefore modified these equations as follows:

$$\begin{array}{l} \mathrm{eq}{.A)\mathrm{ARGSUC}‒>\mathrm{ARG}+4\mathrm{CO}}_{2}{‒6\mathrm{NAD}}^{+}{+6\mathrm{NADH}‒2\mathrm{FAD}}^{+}\\ \;+2\mathrm{FADH}‒2\mathrm{ADP}‒2\mathrm{Pi}+2\mathrm{ATP}; \end{array}$$

$$\begin{array}{l} \mathrm{eq}{.B)\mathrm{SAICAR}‒>\mathrm{AICAR}+4\mathrm{CO}}_{2}{‒6\mathrm{NAD}}^{+}{+6\mathrm{NADH}‒2\mathrm{FAD}}^{+}\\ \;+2\mathrm{FADH}‒2\mathrm{ADP}‒2\mathrm{Pi}+2\mathrm{ATP} \end{array}$$

These modifications resolved the biological conundrum caused in our previous model by the negative stoichiometry coefficients of OAA and FUM in the biomass composition. For glycogen production, we modified the stoichiometry as follows:

1glycogen=8G6P+8ATP

[37].

In our previous model, stoichiometric coefficients indicating the relation between key precursors for biomass production and other general metabolites were not integers. One of the reasons for this phenomenon is the use of a pseudoinverse matrix in the MATLAB script. For example, the balance equation for S7P was expressed as follows:

eq.C)1S7P=0.5E4P‒1GAP+0.5R5P+0.5F6P+0.5X5P

In our model, S7P appeared in 2 reactions:

1E4P+1F6P=1GAP+1S7P

and

1X5P+1R5P=1GAP+1S7P

When we individually transformed these reactions, the 2 reactions were expressed as follows:

1S7P=‒1GAP+1E4P+1F6P

and

1S7P=‒1GAP+1R5P+1X5P,

in which the coefficients were integers. However, when we combined these 2 equations into a single equation, we calculated the weighted average; thus the stoichiometry of the mass balance equation for S7P is not an integer, as shown in eq. C. In our MATLAB script, we transformed these reactions using a pseudoinverse matrix, thereby obtaining equivalent average coefficients. These non-integer coefficients are caused by metabolites that are at the branch-points of a metabolic reaction. To remove these apparent defects, we added metabolites at branch-points, like S7P, to the key substances required for biomass production. In this regard, however, these modifications were made merely for purposes of calculation and do not influence biomass production.

### Calculation, sensitivity, and scale factor

We used the ode15s function in MATLAB for simulations over a calculation period of 840 minutes with 5 min intervals. The sensitivity analysis (results shown in Table 1) was performed as follows. Maximum sensitivity was defined as the maximum value for the ratio of l-glutamic acid yield to the variation in gene copy number or enzyme concentration. The initial l-glutamic acid yield was defined as Y0, and the l-glutamic acid yield obtained from the parameter-modified model was defined as Y. By changing each parameter and by multiplying with 0.001 to 1000 at an exponential rate, a maximum value of (Y – Y0)/Y0 was explored. Thus, maximum sensitivity was defined as maximum value of (Y – Y0)/Y0. The scale factor was defined as the model parameter that resulted in maximum sensitivity (Table 1). The initial model parameter was defined as X0, and the modified parameter, giving the maximum sensitivity, was defined as X; the scale factor was defined as X/X0.

### Strains, plasmids, and medium

The strains and plasmids used in this study are shown in Table 4. For *pgk* cloning, primers with the sequences 5′-gcggatccctgtaaaagccaatgaatgtc-3′ and 5′-gcaagcttattacgccaggttttacgaa-3′ were used to amplify *pgk* from *E. coli* genomic DNA. After *Bam*HI and *Hin*dIII digestion of the PCR fragment, it was cloned into pUC118 (Takara Bio.). *E. coli* DH5α was used as the cloning host and *E. coli* MG1655 Δ*sucA* was used as the l-glutamic acid fermentation strain. To construct the pTWV228-P_*tac*_-T_*trp*_ vector, the synthesized *tac* promoter region and tryptophan operon terminator region were cloned into *Bam*HI- and *Kpn*I-digested pTWV228 (Takara Bio) vector. For *gltA* cloning, primers with sequences 5′-cacaaggagactcccatggctgatacaaaagcaaaactc-3′ and 5′-gaactggcggctcccttaacgcttgatatcgcttttaaa-3′ were used to amplify *gltA* from *E. coli* genomic DNA. For *icdA* cloning, primers with sequences 5′-cacaaggagactcccatggaaagtaaagtagttgttccg-3′ and 5′-gaactggcggctcccttacatgttttcgatgatcgcgtc-3′ were used to amplify *icdA* from *E. coli* genomic DNA. By using an in-fusion cloning kit (Clontech), these fragments were cloned into a *Sma*I-digested pTWV228-P_*tac*_-T_*trp*_ vector to construct the pTWV228-P_*tac*_-*gltA*-T_*trp*_ and pTWV228-P_*tac*_-*icdA*-T_*trp*_ expression vectors, respectively. Shake-flask culture was used for fermentation. The composition of the medium for l-glutamic acid fermentation was as follows: 40 g/L of glucose, 1 g/L of MgSO_4_·7H_2_O, 20 g/L of (NH_4_)_2_SO_4_, 1 g/L of KH_2_PO_4_, 10 mg/L of FeSO_4_·7H_2_O, 10 mg/L of MnSO_4_·7H_2_O, 2 g/L of yeast extract, and 0.6 g/flask of CaCO_3_. Chloramphenicol (30 mg/L), Tetracycline (25 mg/L) or ampicillin (100 mg/L) was added to the media as required to select for the corresponding markers in the bacterial chromosome or plasmid. Residual glucose and l-glutamic acid concentrations were measured by using an enzymatic electrode, using BF-5 (Oji Keisokuki).

**Table 4:** Strains and plasmids

**Strain**	**Reference**
*E. coli* MG1655 Δ*sucA*	[9]
*E. coli* DH5α	TOYOBO
**Plasmid**	**Reference**
RSF-PPG	[28]
pUC118	TAKARA BIO
pUC118-*pgk*	this study
pTWV228	TAKARA BIO
pTWV228-P_*tac*_-T_*trp*_	this study
pTWV228-P_*tac*_-*gltA*-T_*trp*_	this study
pTWV228-P_*tac*_-*icdA*-T_*trp*_	this study

## Abbreviations

13DPG: 1,3-bisphosphoglycerate; 2PG: 2-phosphoglycerate; 3PG: 3-phosphoglycerate; 6PGD: 6-phosphogluconate dehydrogenase; AAT: Aspartate aminotransferase; ACCoA: Acetyl coenzyme A; aceA: Gene encoding isocitrate lyase; aceB: Gene encoding malate synthase; aceE: Gene encoding a subunit of the E1p component of pyruvate dehydrogenase complex; aceF: Gene encoding a subunit of the E2 component of pyruvate dehydrogenase complex; aceK: Gene encoding isocitrate dehydrogenase kinase/phosphatase; ACNA: Aconitase; acnA: Gene encoding aconitase; ACNB: Aconitase; acnB: Gene encoding aconitase; ACOH: Acetate; ACOHxt: External acetate; ADP: Adenosine 5′-diphosphate; AICAR: 5-aminoimidazole-4-carboxamide ribotide; AKG: α-ketoglutarate; AMP: Adenosine monophosphate; ARG: Arginine; ARGSUC: Arginosuccinate; ASP: Aspartate; aspC: Gene encoding aspartate aminotransferase; ATP: Adenosine 5′-triphosphate; b2463: Gene encoding malate dehydrogenase; cAMP: Cyclic adenosine monophosphate; cAMPxt: External cyclic adenosine monophosphate; CEX: cAMP efflux protein; CIT: Citrate; CO2: Carbon dioxide; CoA: Coenzyme A; CPD: cAMP phosphodiesterase; cpdA: Gene encoding cAMP phosphodiesterase; Cra: Catabolite repressor/activator; CRP: cAMP receptor protein; crp: Gene encoding cAMP receptor protein; crr: Gene encoding Crr a subunit of the glucose phosphotransferase system; CS: Citrate synthase; CYA: Adenylate cyclase; cyaA: Gene encoding adenylate cyclase; D6PGC: 6-phospho-d-gluconate; DHAP: Dihydroxyacetone phosphate; dPGM: 2,3-bisphosphoglycerate-dependent phosphoglycerate mutase; E4P: Erythrose 4-phosphate; EI: Phosphotransferase system enzyme I; ENO: Enolase; eno: Gene encoding enolase; epd: Gene encoding erythrose 4-phosphate dehydrogenase; F6P: Fructose 6-phosphate; FAD+: Flavin adenine dinucleotide; FADH: Reduced flavin adenine dinucleotide; FBA: Fructose bisphosphate aldolase; fba: Gene encoding fructose bisphosphate aldolase; FBP: Fructose 1,6-bisphosphatase; fbp: Gene encoding fructose 1,6-bisphosphatase; FDP: Fructose 1,6-bisphosphate; FORM: Formate; FORMxt: External formate; FRD: Fumarate reductase; frdA: Gene encoding fumarate reductase flavoprotein; frdB: Gene encoding fumarate reductase iron-sulfur protein; frdC: Gene encoding fumarate reductase membrane protein; frdD: Gene encoding fumarate reductase membrane protein; fruA: Gene encoding fructose phosphotransferase system permease FruA subunit; fruB: Gene encoding fructose phosphotransferase system permease FruB subunit; FRUK: 1-phosphofructokinase; fruK: Gene encoding 1-phosphofructokinase; fruR: Gene encoding catabolite repressor activator; FUM: Fumarate; FUMA: Fumarase; fumA: Gene encoding fumarase; G6P: Glucose 6-phosphate; G6PD: Glucose-6-phosphate-1-dehydrogenase; GA3P: Glyceraldehyde 3-phosphate; GAPA: Glyceraldehyde 3-phosphate dehydrogenase; gapA: Gene encoding glycerate 3-phosphate dehydrogenase; GDH: Glutamate dehydrogenase; gdh: Gene encoding glutamate dehydrogenase; GLCxt: External glucose; gltA: Gene encoding citrate synthase; GLU: Glutamate; GLUxt: External glutamate; GLX: Glyoxylate; gnd: Gene encoding 6-phosphogluconate dehydrogenase; gpmA: Gene encoding 2,3-bisphosphoglycerate-dependet phosphoglycerate mutase; HPr: Histidine protein; ICD: Isocitrate dehydrogenase; icdA: Gene encoding isocitrate dehydrogenase; ICDK: Isocitrate dehydrogenase kinase/phosphatase; ICD-P: Phosphorylated isocitrate dehydrogenase; ICIT: Isocitrate; ICL: Isocitrate lyase; IclR: Acetate operon regulator; iclR: Gene encoding isocitrate lyase regulator; IIAGlc: IIA subunit of glucose phosphotransferase system; IICBGlc: IICB subunit of glucose phosphotransferase system; iPGM: 2,3-bisphosphoglycerate-independent phosphoglycerate mutase; KGDH: α-ketoglutarate dehydrogenase; MAL: Malate; MDH: Malate dehydrogenase; mdh: Gene encoding malate dehydrogenase; Mlc: Making large colony protein; mlc: Gene encoding making large colony protein; MS: Malate synthase; NAD+: Nicotinamide adenine dinucleotide; NADH: Reduced nicotinamide adenine dinucleotide; NADME: Malic enzyme NAD+−linked; NADP+: Nicotinamide adenine dinucleotide phosphate; NADPH: Reduced nicotinamide adenine dinucleotide phosphate; NADPME: Malic enzyme NADP+−linked; OAA: Oxaloacetate; PCK: Phosphoenolpyruvate carboxykinase; pckA: Gene encoding phosphoenolpyruvate carboxykinase; PDH: Pyruvate dehydrogenase; PdhR: Pyruvate dehydrogenase complex regulator; pdhR: Gene encoding pyruvate dehydrogenase complex regulator; PEP: Phosphoenolpyruvate; PEPC: Phosphoenolpyruvate carboxylase; PFKA: 6-phosphofructokinase; pfkA: Gene encoding 6-phosphofructokinase; PGI: Phosphoglycerate mutase; pgi: Gene encoding phosphoglycerate mutase; PGK: Phosphoglycerate kinase; pgk: Gene encoding phosphoglycerate kinase; PGM: Phosphoglycerate mutase; Pi: Phosphate; ppc: Gene encoding phosphoenolpyruvate carboxylase; PPP: Pentose phosphate pathway; PPS: Phosphoenolpyruvate synthetase; ppsA: Gene encoding phosphoenolpyruvate synthetase; ptsG: Gene encoding IICBGlc; ptsH: Gene encoding HPr; ptsI: Gene encoding EI; PYKF: Pyruvate kinase I; pykF: Gene encoding pyruvate kinase I; PYR: Pyruvate; R5P: Ribose 5-phosphate; RL5P: Ribulose 5-phosphate; RPE: Ribulose-5-phosphate 3-epimerase; rpe: Gene encoding ribulose-5-phosphate 3-epimerase; RPI: Ribose-5-phosphate isomerase A; rpiA: Gene encoding ribose-5-phosphate isomerase A; S7P: Sedoheptulose 7-phosphate; SAICAR: 5′'-phosphoribosyl-4-(*N*-succinocarboxamide)-5-aminoimidazole; SCS: Succinyl coenzyme A synthetase; SDH: Succinate dehydrogenase; sdhA: Gene encoding succinate dehydrogenase flavoprotein; sdhB: Gene encoding succinate dehydrogenase iron-sulfur protein; sdhC: Gene encoding succinate dehydrogenase membrane protein; sdhD: Gene encoding succinate dehydrogenase membrane protein; sfcA: Gene encoding malic enzyme NAD+−linked; sucA: Gene encoding a subunit of the E1 component of α-ketoglutarate dehydrogenase complex; sucB: Gene encoding the SucB subunit of the α-ketoglutarate dehydrogenase complex; sucC: Gene encoding succinyl coenzyme A synthetase β subunit; SUCCoA: Succinyl coenzyme A; sucD: Gene encoding succinyl coenzyme A synthetase α subunit; TALB: Transaldolase B; talB: Gene encoding transaldolase B; tktA: Gene encoding transketolase; TKTAI: Transketolase I; TKTAII: Transketolase II; TPI: Triose phosphate isomerase; tpiA: Gene encoding triose phosphate isomerase; X5P: Xylulose 5-phosphate; yibO: Gene encoding 2,3-bisphosphoglycerate-independent phosphoglycerate mutase; zwf: glucose Gene encoding 6-phosphate-1-dehydrogenase.

## Competing interests

The authors declare that they have no competing interests.

## Authors’ contributions

YN carried out the fermentation studies, and SO, MI, TM performed the simulation study. YY, YU, HT participated in the design of the study and helped to draft manuscript. All authors read and approved the final manuscript.
